# Chemoprevention of esophageal adenocarcinoma

**DOI:** 10.1093/gastro/goaa040

**Published:** 2020-07-24

**Authors:** Erik J Snider, Andrew M Kaz, John M Inadomi, William M Grady

**Affiliations:** 1 Division of Gastroenterology, Department of Internal Medicine, University of Washington School of Medicine, Seattle, WA, USA; 2 Gastroenterology Section, VA Puget Sound Health Care System, Seattle, WA, USA; 3 Clinical Research Division, Fred Hutchinson Cancer Research Center, Seattle, WA, USA

**Keywords:** Barrett’s esophagus, esophageal adenocarcinoma, chemoprevention

## Abstract

Esophageal adenocarcinoma (EAC) is a major cause of cancer-related death, particularly in Western populations, and is rapidly rising in Asian populations at this time. Virtually all EACs develop from the precursor lesion Barrett’s esophagus (BE), which is the most significant risk factor for EAC. However, the rates of progression from BE to EAC are low and patients with BE are asymptomatic. Thus, any strategy for EAC prevention must carry a low risk of harm in order to be clinically useful. Since current EAC-screening and BE-surveillance methods carry some procedural risk and are burdensome, there is an opportunity for chemoprevention, i.e. medications or dietary factors that may prevent BE from progressing to EAC. A variety of candidate chemoprevention therapies have been assessed to date. Proton-pump inhibitors (PPIs) are the best studied and have modest EAC-chemoprevention efficacy in BE patients, with a recent randomized trial showing that high-dose PPI may be more effective than low-dose PPI. Aspirin and other non-steroidal anti-inflammatory drugs have moderate quality observational and randomized-trial evidence for preventing progression of BE to EAC, but their risks for harm have precluded their routine clinical use. Other therapies (statins, metformin, female sex hormones) generally do not have strong evidence to support their use in EAC chemoprevention. Although progress has been made in this field, there is still a need for more effective and safe chemoprevention therapies for EAC.

## Introduction

### Barrett’s esophagus and esophageal adenocarcinoma

**Figure 1.**
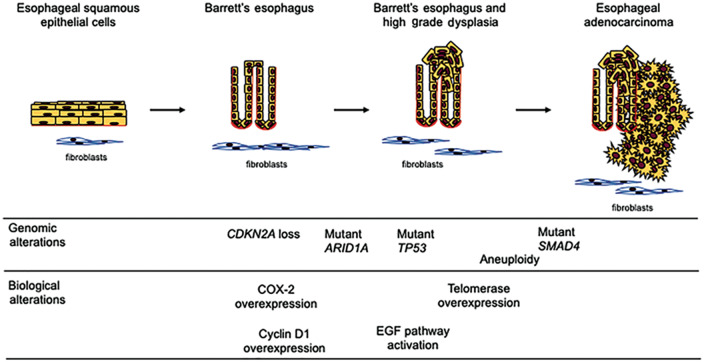
Schematic diagram of the Barrett’s esophagus-to-esophageal adenocarcinoma progression sequence. Normal esophageal epithelium gives rise to specialized intestinal metaplasia (Barrett’s esophagus), which can transform into dysplastic tissue and then cancer. Some of the common genetic and biologic alterations seen during this process are shown. HCl, hydrochloric acid; EGF, epidermal growth factor; COX-2, cyclooxygenase 2.

Esophageal adenocarcinoma (EAC) has shown a dramatic increase in incidence in the last 50 years in Western populations and is rising substantially in developing countries. It currently affects 18,000 people per year in the USA [1–3]. EAC is often asymptomatic until locally invasive, with 5-year case overall survival rates among the lowest of all cancers, despite recent improvements in surveillance and treatment [1, 4]. There is therefore significant interest in predicting and preventing the development of EAC in patients with Barrett’s esophagus (BE), the premalignant precursor of virtually all EACs (Figure 1).

BE affects 1%–3% of people in Western societies and is found most commonly in individuals with certain risk factors [1]. Chronic gastroesophageal reflux disease (GERD) is the strongest single clinical risk factor for the development of BE and/or EAC [5]. Caucasian race, male gender, obesity, advanced age (>60 years), tobacco use, and alcohol use are other well-recognized risk factors [6]. *Helicobacter pylori* infection is associated with a reduced risk of BE [1, 6, 7].

Although BE is relatively common, progression of BE to EAC is low, with recent epidemiologic studies showing rates from 0.1% to 0.5% of non-dysplastic BE patients per year being diagnosed with EAC [8–10]. Importantly, because BE is asymptomatic, the vast majority of BE carriers are unaware of having BE, which has led to the advent of BE-screening programs as a way to identify patients at high risk of progression to EAC. Although there is controversy surrounding the clinical utility and cost-effectiveness of BE screening and surveillance, current society guidelines recommend endoscopic-based screening of individuals at high risk of BE and EAC and endoscopic surveillance of those people with BE [11–13]. The risk of progression of BE is higher in people with increasing degrees of dysplasia detected in esophageal biopsies of the BE tissue [4]. Therefore, current guidelines recommend that patients with dysplasia undergo more frequent surveillance exams and that individuals are offered endoscopic eradication therapy (radiofrequency ablation ± endoscopic mucosal resection) if high-grade dysplasia is found [11].

Although effective, this strategy carries the risk of procedural complications and has a high healthcare-cost burden because of the use of frequent endoscopic exams [14]. The relatively high prevalence of BE but low progression rate of BE to EAC coupled with the costs, semi-invasive nature, and inconvenience of endoscopy-based screening and surveillance methods has created a need both for non-invasive, inexpensive, accurate, and convenient screening technologies and for chemoprevention therapies. Because advanced EAC carries a poor prognosis, effective prevention of EAC is highly desirable, but any preventative measure must either carry minimal clinical risk to the general population or be applied only to selected high-risk populations in order to have an acceptable risk-to-benefit ratio.

### Chemoprevention

The use of medications or supplements for the prevention and/or mitigation of risk of disease (i.e. chemoprevention) is well established across many fields of medicine. For example, aspirin is commonly used for either the primary or secondary prevention of the development of atherosclerotic coronary vascular disease and its complications [15]. Well established roles for cancer chemoprevention exist for the use of selective-estrogen receptor modulation in women at high risk of breast cancer or for the use of 5-alpha reductase inhibitors in men at high risk of prostate cancer [16, 17]. Chemoprevention also has a potential role in the prevention of certain gastrointestinal cancers;aspirin has shown a protective benefit against the development of colon cancer in populations at risk [18, 19].

Patients with BE frequently have symptomatic GERD and require treatment with acid-suppressive medications such as proton-pump inhibitors (PPIs). They are also frequently prescribed medications for other co-morbid conditions such as coronary artery disease or diabetes because of the high prevalence of these conditions in elderly, obese, Caucasian males [20]. Medications prescribed for such common conditions may have potential anticancer mechanisms of action and have potential to be used as chemoprevention agents, in part because their safety has already been demonstrated by their approval for clinical use. Epidemiological and cross-sectional studies have described associations of several of these medications with a reduced risk of esophageal adenocarcinoma, although few randomized trials have been performed to determine the clinical efficacy of these medications when used prospectively to prevent BE and EAC. This review will summarize the current evidence from observational cohort studies, case–control studies, and clinical trials for the use of medications to reduce the risk of EAC in patients with BE.

## Candidate chemoprevention therapies for esophageal adenocarcinoma in BE patients

### Agents that suppress gastric hydrochloric acid

#### PPIs

**Figure 2.**
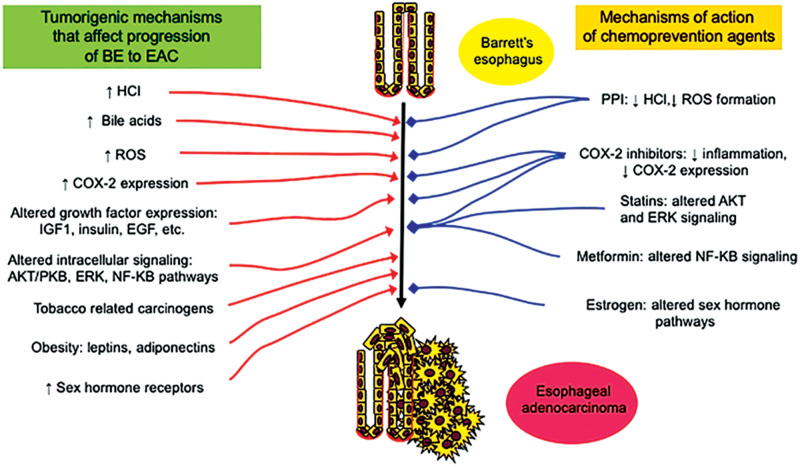
Barrett’s esophagus-to-esophageal adenocarcinoma tumorigenic factors and mechanisms of action of chemoprevention agents. Chemoprevention agents act to potentially inhibit a variety of mechanisms that contribute to carcinogenesis of esophageal adenocarcinoma. HCl, hydrochloric acid; PPI, proton-pump inhibitor; COX-2, cyclooxygenase 2; ROS, reactive oxygen species; IGF, insulin-like growth factor; EGF, epidermal growth factor; AKT/PKB, protein kinase B; ERK, extracellular signal-related kinases; NF-KB, nuclear factor kappa-light-chain-enhancer of activated B-cells; HER2, human epidermal growth factor receptor 2.

Reflux of acidic and bilious stomach contents into the lower esophagus leads to chronic inflammation and the formation of reactive oxygen species, which have been shown to contribute to the carcinogenesis of EAC [21–23]. It therefore follows that raising the pH of the stomach or preventing the reflux of gastric fluid should prevent at least some of these effects and may have a cancer-preventing effect in the clinical setting. (Candidate chemopreventive mechanisms for the PPIs as well as other agents are shown in Figure 2.)

The preponderance of early studies of PPIs in patients with BE or EAC favored a protective effect of PPIs on the development of EAC [24]. Published data from the mid-2000s to early 2010s consistently showed a reduced odds ratio (OR) of high-grade dysplasia (HGD)/EAC in patients using PPIs, with meta-analyses at that time demonstrating a 71% risk reduction in HGD/EAC in BE patients using PPIs (OR 0.29, 95% confidential interval [CI] 0.12–0.79) [24–30]. However, two recently published high-quality observational trials conflict with this prior data, casting doubt on this premise. Hvid-Jensen *et al.* [31], in their case–control study of 1,440 patients with BE from a national cohort in Denmark, demonstrated an increased OR of EAC in patients on PPIs (OR 2.39, 95% CI 1.03–5.54). Masclee *et al.* [32] also studied a large national cohort of BE patients in the UK and observed an increased odds ratio of HGD/EAC in patients using PPIs (OR 1.95, 95% CI 1.00–3.81). Proposed reasons for these conflicting results compared with other published studies include differences in the epidemiology and risk factors in American vs European patients with BE and the possibility of confounding by indication (e.g. patients with more symptomatic or severe reflux were more likely to be prescribed PPIs) [24]. A more recent meta-analysis, including the recent negative studies as well as previous positive data, demonstrated a trend toward a protective effect of PPIs on the development of HGD/EAC, but the effect no longer reached statistical significance (OR 0.43, 95% CI 0.17–1.08) [33].

Adding to the uncertainty and controversy surrounding the use of PPIs for chemoprevention of BE/EAC, data from a recent randomized trial supports the use of PPIs for chemoprevention of EAC. The AspECT trial randomized 2,557 patients with confirmed BE to high- vs low-dose PPI therapy (esomeprazole 40 mg twice daily vs 20 mg once daily). At 8.9 years (mean follow-up), high-dose PPI was superior to low-dose PPI in the primary composite endpoint of time to mortality or development of HGD or EAC (time ratio 1.27, 95% CI 1.01–1.58) [34].

Notably, PPIs are currently the only medications for the prevention of progression of BE that are included in clinical guidelines, with recommendations for PPI use at least once daily in all BE patients, although the evidence supporting this recommendation is not felt to be strong [11, 13]. From a practical standpoint, high-dose PPI use in BE patients is common in clinical practice; however, this is currently only recommended in BE patients with breakthrough symptoms on low-dose PPIs. Given that these guidelines were published before results of the AspECT trial were available, it remains to be seen whether a strategy of ‘minimal effective dosing’ will continue to be recommended in individuals with BE for the management of GERD and HGD/EAC chemoprevention. An important consideration if implementing a high-dose PPI strategy in BE patients includes the associated medication costs and potential dose-related adverse effects from chronic gastric acid suppression, including infectious complications (pneumonia; *Clostridium difficile*), micronutrient deficiencies, osteoporosis, and renal insufficiency [35, 36]. These issues are important for clinicians and population health experts to consider when determining the risks vs benefits of a chemoprevention strategy for BE.

#### Histamine-2 receptor antagonists

Histamine-2 receptor antagonists (H2RAs) are the other major class of medication used in the treatment of GERD, but they have not been shown to affect the rate of neoplastic progression in observational trials of BE. Thota *et al.* [37] demonstrated in a single-center cohort that H2RA use was not protective against HGD/EAC (RR 0.62 95% CI 0.27–1.41), which is generally consistent with previously published trials [24, 28, 30], although some studies have shown a protective effect [38]. Additionally, H2RAs do not appear to have any synergistic protective effect when combined with PPIs [30]. This lack of chemoprotective effect from H2RAs has been attributed to the development of tachyphylaxis and therefore a lack of long-term acid-suppressive benefit. H2RAs are currently not recommended for risk reduction or chemoprophylaxis in BE patients in clinical guidelines [11, 13].

### Aspirin and non-steroidal anti-inflammatory drugs

Aspirin and many other non-steroidal anti-inflammatory drugs (NSAIDs) have been associated with a reduced risk of many gastrointestinal-tract cancers, including cancers of the upper gastrointestinal tract [39]. Given their ready availability as a generic over-the-counter medication, there has been substantial interest in their chemoprevention effects. Notably, patients with BE are often prescribed long-term aspirin for co-morbid cardiovascular conditions. Aspirin use has been associated with a decreased risk of HGD/EAC in several observational trials, as described below.

Cyclooxygenase 2 (COX-2)-mediated inflammation has been shown to be associated with the progression of BE metaplasia to dysplasia [40]. Aspirin and other NSAIDs are cyclooxygenase inhibitors and reduce tissue levels of pro-tumorigenic prostaglandins, such as prostaglandin E2 (PGE2). They have been shown to inhibit the progression of BE in preclinical trials and animal models through this mechanism [41, 42]. Aspirin also modulates the carcinogenic activation of nuclear factor kappa-light-chain-enhancer of activated B-cells (NF-κB) and CDX2 expression in patients with BE [43]. Aspirin use has been well studied for chemoprevention of colorectal cancer and its use for this indication is recommended by the US Preventative Task Force for adults aged 50–59 years, although this depends on a calculation of the patient’s cardiovascular risk and concurrent need for cardiovascular disease risk reduction [44–46].

In a 2014 meta-analysis of four studies comprising 2,152 patients from the USA, the UK, and the Netherlands, Zhang *et al.* [47] showed a protective effect of aspirin on the risk of HGD/EAC in BE patients (RR 0.63, 95% CI 0.43–0.94). Based on these data, a cost-effectiveness analysis demonstrated that the addition of aspirin chemoprevention to regular endoscopic surveillance would result in both decreased healthcare costs and improved quality-adjusted life years for BE patients [48]. However, results of observational data since that time have not shown consistent protective effects of aspirin on EAC risk. Masclee *et al.* [32], in their nested case–control study of a large cohort of BE patients from the Netherlands and the UK, showed no benefit of either long-term or any use of low-dose aspirin (OR 0.9, 95% CI 0.4–1.8; OR 0.9, 95% CI 0.4–2.1). In patients diagnosed with EAC, aspirin use had no effect on mortality [49]. It is also important to recognize that regular aspirin use carries a significant clinical risk of gastrointestinal and cerebral hemorrhage [50], but the nature of the observational trials performed to date has not allowed an analysis of these risks.

The AspECT trial, as described above in the discussion of PPI use, also randomized participants into aspirin and non-aspirin use, excluding patients with a contraindication to aspirin use or who were already prescribed aspirin for cardiovascular indications [34]. Overall, individuals receiving aspirin (300 mg daily) had a trend toward improved time to mortality or development of HGD/EAC that did not reach statistical significance (TR 1.24, 95% CI 0.98–1.57). However, in the subgroup of patients not already taking other NSAIDs, aspirin use did achieve a statistically significant protective effect (TR 1.29, 95% CI 1.01–1.66). Additionally, aspirin use appeared to provide additive benefits when combined with high-dose PPI as compared with low-dose PPI and no aspirin (TR 1.59, 95% CI 1.14–2.23). Patients in the aspirin-therapy arm had higher rates of serious gastrointestinal hemorrhage than patients taking concurrent PPI therapy with no aspirin, but overall numbers of events were very low (*n *=* *14 vs *n *=* *6, *P* not reported).

Non-aspirin NSAIDs are often included in studies investigating aspirin and have a similar purported mechanism of action. However, given that NSAIDs are generally less likely to be prescribed for long-term use and are frequently associated with unrecorded, over-the-counter use, the effect of these medications is challenging to evaluate in retrospective analyses. A meta-analysis published in 2014 showed a relative risk of 0.50 for HGD/EAC (95% CI 0.32–0.78) in NSAID users, but the frequency of use, dosage, and magnitude of protective effect varied widely among studies [47]. Masclee *et al.* [32], when examining NSAIDs in their cohort, did not show a protective effect on the progression of BE to HGD/EAC (OR 0.9, 95% CI 0.5–1.8). In contrast, in a prospective cohort of 570 Dutch patients, non-aspirin NSAIDs did demonstrate a protective effect against BE progression that was independent of aspirin use (HR 0.47, *P *=* *0.03) [51].

One small randomized trial of celecoxib in 100 patients with low-grade dysplasia or HGD showed no benefit in terms of dysplasia progression after 48 weeks [52]. Because chronic non-aspirin NSAID use is associated with significant clinical adverse events (peptic ulcer disease, acute or chronic kidney disease, etc.) without any of the positive cardioprotective effects of aspirin, in the absence of higher-quality evidence, they are not recommended for routine use for EAC chemoprevention [13].

### Statins

HMG-coA reductase inhibitors, or statins, are a medication class commonly prescribed for the treatment of dyslipidemia in patients at risk of cardiovascular disease. Because of the common co-occurrence of BE and coronary artery disease risk factors, the impact of HMG-coA reductase inhibitors on BE can be readily assessed in observational cohort studies. Statin use has been shown to be associated with reduced overall cancer mortality and multiple observational studies have shown a reduced incidence of EAC in statin users [28, 32, 53].

The anti-carcinogenic effect of statins does not appear to be dependent solely on cholesterol-biosynthesis pathways. *In vitro* studies of statins demonstrated inhibition of proliferation and induction of apoptosis in EAC cell lines through the extracellular signaling regulated kinase (ERK) and AKT signaling pathways, as well as through the enzyme-dependent farnesylation of cholesterol precursors [54]. Statins have also been shown to decrease the malignant potential of EAC cells in *in vitro* cell-line studies through decreasing the expression of intracellular adhesion molecule 1 (ICAM-1) [55].

A 2017 meta-analysis of 11 studies, comprising almost 19,000 patients with BE, showed an OR of 0.59 of EAC among statin users, with consistent effects seen across all included studies (95% CI 0.50–0.68) [56]. This benefit might be dose-related, as Beales *et al.* [57] demonstrated a reduced OR of EAC in patients taking high-dose statin therapy (equivalent to simvastatin 40 mg daily or higher) compared with that in those taking low-dose therapy (OR 0.31, 95% CI 0.05–0.97 vs OR 0.54, 95% CI 0.27–0.98).

The duration of therapy required to benefit from statins is unclear at this time, although increased time of exposure has been shown to correlate with a greater magnitude of protective effect across multiple studies [28, 32, 58]. The protective effect of statins on EAC incidence appears to be potentiated by COX-2 inhibition, with additive effects seen with both aspirin and non-aspirin NSAIDs use [51, 57]. At this time, prospective clinical trials are needed to assess the efficacy of these agents in populations with BE.

### Metformin

Metformin is a medication that decreases hepatic glucose production, decreases intestinal absorption of glucose, and improves insulin sensitivity by increasing peripheral glucose uptake and utilization. It is commonly prescribed for the treatment of adult-onset diabetes mellitus and has demonstrated protective anti-carcinogenic effects for a variety of gastrointestinal cancers, particularly colorectal cancer [59]. Metformin has also been shown to reduce the growth of EAC xenografts and suppress tumor formation in mouse models of EAC through reduced activation of a wide variety of oncogenes, including epidermal growth factor receptor, vascular endothelial growth factor, and insulin-like growth factor (IGF), and by inhibiting the phosphorylation of S6 kinase 1 (S6K1), which mediates the mTOR signaling pathway [60]. However, observational population-based studies on the association of metformin with EAC incidence are limited and generally have demonstrated no association with reduced EAC risk. A retrospective analysis of 138 male BE patients with type II diabetes did not show any significant association of metformin use with the risk of EAC (*P *=* *0.138) [61]. Furthermore, a small randomized trial of 74 patients with BE given metformin or placebo did not show any difference in its primary endpoint of a reduction in tissue pS6K1 levels at 12 weeks, suggesting that metformin would not inhibit the progression of BE to EAC [62].

### Sex hormones

The high male-to-female ratio of BE and EAC incidence, which is not entirely explained by differences in the prevalence of other clinical risk factors for EAC, has led to the hypothesis that female sex hormones may be protective against the development of EAC [63]. Exogenous estrogen use most commonly occurs in postmenopausal women using hormone replacement therapy (HRT) or in premenopausal women using oral contraceptives. A number of small population-based studies of women taking exogenous estrogens have demonstrated a trend towards a protective effect against EAC. A meta-analysis of these studies showed a slight statistically significant protective effect against EAC of HRT and a trend toward protection with oral contraceptives in premenopausal women (OR 0.75, 95% CI 0.58–0.98 and OR 0.76, 95% CI 0.57–1.00) [64]. The largest retrospective analysis of the effect of HRT on EAC utilized the Women’s Health Initiative database of 160,080 postmenopausal women and assessed the incidence of EAC in a subset of the subjects who were randomized to HRT. In this cohort, there was no statistically significant association between a diagnosis of EAC and hormone use (either estrogen or estrogen plus progesterone) [65]. This study, like others investigating the risk of EAC in female patients, had relatively small total case numbers, with only 23 total cases of EAC in the cohort, which limits the power to detect small effect sizes. Data from studies specifically evaluating the effect of estrogens on EAC incidence in previously diagnosed female BE patients are currently lacking.

### Other candidate prevention agents

A multitude of other medications and dietary agents have been assessed in preclinical studies and small clinical trials for their potential roles for EAC chemoprevention, but there is generally a lack of significant clinical data to support their use at this time. Some of the more promising agents and medications will be discussed in this section.

Difluoromethylornithine (DFMO) is an inhibitor of ornithine decarboxylase (OTC), which has been shown to promote oncogenesis through its effects on polyamine metabolism in preclinical studies [66]. High levels of OTC and polyamines have been shown to be expressed in BE and EAC tissues [67]. A single-arm trial of 10 non-dysplastic BE patients given DFMO showed reduced levels of tissue polyamines after 6 months, but studies measuring EAC development in BE patients given DFMO are lacking at this time [68].

Studies assessing the association of vitamin D deficiency with EAC have provided conflicting evidence for the potential role of vitamin D for EAC chemoprevention. Some observational studies have shown an association between vitamin D deficiency and an increased risk of EAC, whereas others have found no such association [69, 70]. A small, non-randomized prospective trial of 18 BE patients did not demonstrate any change in mucosal expression of a set of tumor-suppressor genes after 12 weeks of high-dose vitamin D supplementation [71].

Ursodeoxycholic-acid supplementation has been hypothesized to alter the acid and bile composition of gastroesophageal refluxate and its use in preclinical studies has shown reduced rates of EAC in a rat model of BE [72]. However, a non-randomized pilot study of 29 patients with BE showed altered bile-acid composition after 6 months of ursodeoxycholic-acid supplementation but no change in the primary study endpoint of altered tissue biomarkers indicative of oxidative damage and apoptosis [73].

Omega-3 polyunsaturated fatty acids have been shown to reduce systemic inflammation through COX-2-mediated mechanisms, which may play a role in EAC carcinogenesis [74, 75]. A small randomized trial of omega-3 fatty-acid supplementation in 52 BE patients showed a reduction in tissue COX-2 protein levels but no reduction in the tissue levels of inflammatory cytokines, such as PGE2 and LTB4 [76].

Folate, given its role in DNA synthesis and repair, has been proposed to play an anti-carcinogenic role in EAC. Observational population-based trials have shown an inconsistent association between dietary folate intake or serum folate levels and reduced incidence of esophageal adenocarcinoma, but no specific trials of folate supplementation in BE patients have been conducted to date [77].

Finally, curcumin, an active phenol derivative of the spice turmeric, has been hypothesized to have anti-carcinogenic effects and has demonstrated *in vitro* activity against oncogenic signaling pathways active in BE progression, such as NF-κB; however, no clinical trial of human curcumin supplementation has been conducted at this time [78].

## Conclusions

BE is relatively common and is the major risk factor for EAC. In light of the high mortality rates of advanced EAC, BE screening and surveillance are currently the standard of care and recommendation by organizations like the American Gastroenterological Association. However, the clinical effectiveness of screening and surveillance in BE patients for preventing EAC is not clear at this time and is a source of controversy. This has led to interest in other approaches for preventing EAC in individuals with BE, including chemoprevention. Barriers to the use of chemoprevention agents have included not only the lack of a therapy with substantial clinical effectiveness, but also a need for agents that have very low or no potential for adverse effects. The rates of progression of BE to EAC are low enough that careful consideration must be paid to the side effects and medical risks of any strategy of chemoprevention.

Endoscopic ablative strategies are now recommended for patients with low-grade or HGD and have been proved to be very effective for eradicating dysplastic tissue, which has relegated the use of primary chemoprevention to non-dysplastic BE. Of interest, because BE does recur and EAC does develop in some patients after endoscopic ablation, the role of chemoprevention after ablation therapy is also under investigation at this time [13].

Despite recent controversy regarding their efficacy, PPIs remain the mainstay for the chemoprevention of EAC for patients with BE. The results of the AspECT trial suggest, contrary to current recommendations, that aspirin and high-dose PPIs are beneficial for patients with non-dysplastic BE, but further studies are needed before they can be recommended for routine clinical use. Statins also deserve ongoing consideration for Barrett’s chemoprevention, although, at this point, their role is limited to those patients who also carry a cardiovascular indication for their use. Overall, PPIs and aspirin appear to have the greatest potential for the chemoprevention of EAC. However, only PPIs are generally recommended for BE patients at this time, and the evidence for this recommendation is modest. In summary, there remains a need for effective and safe chemoprevention agents for EAC and ongoing investigation of promising but clinically unproven prevention therapies.

## Authors’ contributions

WG: study concept and design, drafting of manuscript, revision of manuscript. ES: study concept and design, drafting of manuscript, revision of manuscript. JI: critical revision of manuscript for important intellectual content. AK: critical revision of manuscript for important intellectual content.

## Funding

Support for this work was provided by National Institutes of Health (NIH) National Cancer Institute (NCI) [RO1CA220004, RO1CA194663, P30CA15704, U01086402, UO1CA152756, U54CA163060, and P01CA077852] (to W.M.G.) and the Cottrell Family Fund and the Listwin Family Foundation.

## Conflicts of interest

WM Grady is on the advisory boards of Guardant Health, Freenome and SEngine and is a consultant for Diacarta. He is also an investigator for a clinical trial sponsored by Janssen Pharmaceuticals and receives services for investigator inititated research from Tempus and Lucid Technologies.
